# Human longevity: 25 genetic loci associated in 389,166 UK biobank participants

**DOI:** 10.18632/aging.101334

**Published:** 2017-12-06

**Authors:** Luke C. Pilling, Chia-Ling Kuo, Kamil Sicinski, Jone Tamosauskaite, George A. Kuchel, Lorna W. Harries, Pamela Herd, Robert Wallace, Luigi Ferrucci, David Melzer

**Affiliations:** ^1^ Epidemiology and Public Health Group, University of Exeter Medical School, RILD Level 3, Royal Devon & Exeter Hospital, Exeter, EX2 5DW, UK; ^2^ Department of Community Medicine and Health Care, Connecticut Institute for Clinical and Translational Science, Institute for Systems Genomics, University of Connecticut Health Center, CT 06269 USA; ^3^ Center for Demography of Health and Aging, University of Wisconsin, Madison, WI 53706, USA; ^4^ UConn Center on Aging, University of Connecticut, Farmington, CT 06030, USA; ^5^ Institute of Biomedical and Clinical Sciences, University of Exeter Medical School, RILD Level 3, Royal Devon & Exeter Hospital, Exeter, UK; ^6^ La Follette School of Public Affairs and the Department of Sociology, University of Wisconsin, Madison, WI 53706, USA; ^7^ College of Public Health, University of Iowa, Iowa City, IA 52242, USA; ^8^ National Institute on Aging, Baltimore, MD 21224, USA

**Keywords:** longevity, GWAS, human, genetic, 1417

## Abstract

We undertook a genome-wide association study (GWAS) of parental longevity in European descent UK Biobank participants. For combined mothers' and fathers' attained age, 10 loci were associated (p<5*10^−8^), including 8 previously identified for traits including survival, Alzheimer's and cardiovascular disease. Of these, 4 were also associated with longest 10% survival (mother's age ≥90 years, father's ≥87 years), with 2 additional associations including *MC2R* intronic variants (coding for the adrenocorticotropic hormone receptor). Mother's age at death was associated with 3 additional loci (2 linked to autoimmune conditions), and 8 for fathers only. An attained age genetic risk score associated with parental survival in the US Health and Retirement Study and the Wisconsin Longitudinal Study and with having a centenarian parent (*n*=1,181) in UK Biobank. The results suggest that human longevity is highly polygenic with prominent roles for loci likely involved in cellular senescence and inflammation, plus lipid metabolism and cardiovascular conditions. There may also be gender specific routes to longevity.

## INTRODUCTION

For centuries there has been great interest in factors enabling some individuals to live to very old ages. Extreme longevity can be achieved in animal models by knocking out many specific genes [1]. In humans there is a growing number of inherited (germline) genetic variants linked to overall [2] and longer human lifespan [3,4] in conventional genome wide association studies (GWAS), and in studies weighted for the large numbers of previously reported variant associations with related diseases and traits [5, 6].

Human longevity or lifespan has moderate heritability in twin studies, estimated at 20-30% [7], with health related behaviors, environmental exposures and chance likely explaining much of the remaining variability. GWAS have mostly studied general survival or compared longer lived individuals (e.g. aged 80+, 90+ or centenarians) to younger controls. An alternative approach to studying older individuals themselves is to study parental longevity. Biological offspring of longer lived parents inherit a combination of parental genetic variants, and tend to remain healthier and live longer than the offspring of shorter lived parents [8]. In the US Health and Retirement Study (HRS), we found that mortality in offspring declined progressively with later parental ages of death [9]. In addition, offspring of longer lived parents had progressively lower incidence of cardiovascular disease and cancers, plus reduced rates of cognitive impairment [10]. These findings were replicated in 186,151 non-adopted UK Biobank participants [11]. Statistical associations of mothers' and fathers' attained ages with offspring health outcomes in HRS were additive. These findings suggest that combined parental longevity is a quantitative trait of slower aging, likely with a significant genetic component.

We [12] and separately Joshi et al. [13] previously undertook a GWAS of parental longevity in the first one third UK Biobank (UKB) participants aged 55-70 [12]. We determined the percent of variance of parental age at death explained by the directly genotyped variants on the UKB arrays (n=845,997): the variance attributed was greatest for the sum of normalized mother's and father's age at death (10.2%, SD=1.26%) and smaller for mothers (6.08% SD=1.21%) and fathers (5.79% SD=1.23%) separately.

In the current analysis we aimed to extend our previous GWAS to include data from the newly available fully genotyped UKB cohort, essentially tripling our previous sample size. We aimed to identify all common genetic variants (prevalence ≥0.1%) associated with longer parental lifespan. We studied combined parents' attained ages but also tested associations with both parents having longest 10% survival, to establish whether variants were associated with long life rather than early death. We also examined mothers' and fathers' age of death separately. We also tested a combined genetic risk score of our findings in two US aging cohorts, the HRS and the Wisconsin Longitudinal Study (WLS).

## RESULTS

Subjects meeting inclusion criteria (Table 1) were 389,166 UK Biobank participants of European descent with data recorded on parents' current ages or parents' ages of death (n=181,048 had one or more living parents). GWAS analyses had >99% power to detect an allele of 1% minor allele frequency, accounting for 0.1% of the variance in the phenotype, after correction for multiple statistical testing (alpha=5×10^−8^). Variants with minor allele frequencies of >0.1 were included.

**Table 1:** Summary statistics for 389,166 UK Biobank participants of European descent with genetic and parental age data

		n	min - max	mean (SD)
Age of study participant (years)		389,166	40 - 73	56.66 (8)
BMI (Kg/m^2^)		387,951	12.12 - 74.68	27.31 (4.73)
Mother's age at death (years)		246,941	56 - 107	77.39 (9.77)
Mother's age if alive (years)		165,996	60 - 105	78.58 (8.15)
Father's age at death (years)		317,652	46 - 106	72.18 (11.05)
Father's age if alive (years)		97,659	60 - 103	78.02 (7.37)
Combined parents' age at death				
	Years (average)	208,118	51 - 101	75.41 (7.79)
	Sum of z-scores	208,118	−3.12 - 3.31	0.01 (1)
Combined parents' age (alive or dead)				
	Years (total)	389,166	102 - 202	151.57 (14.98)
		**n**	**%**	
Sex				
	Females	212,672	54.65	
	Males	176,494	45.35	
Smoking status				
	Never	212,833	54.87	
	Former	136,647	35.23	
	Current	38,414	9.9	
Highest education level attained				
	None	62,519	16.21	
	Secondary	65,585	17	
	College-level	71,048	18.42	
	Professional/University	186,572	48.37	
Both parents top 10% survival				
	No (both died <80)	79,767	91.74	
	Yes (≥90 mothers, ≥87 fathers)	7,182	8.26	
Combined parents' age (alive or dead)				
	Either alive	181,048	46.52	
	Both dead	208,118	53.48	

### GWAS of parental attained age

We examined associations with both parents' combined attained ages (including ages of living parents), using Martingale residuals from Cox's proportional hazards regression models, following the approach used by Joshi et al. [13]. In this analysis we found 10 loci associated at genome wide significance (p<5×10^−8^) (Table 2; Supplementary Table 1 for details of all significant variants; full results available to download https://doi.org/10.6084/m9.figshare.5439382.v1).

**Table 2:** GWAS associations with parents' attained age phenotypes

							P-values for phenotype:					Effect for the analysis in bold			
		Lead SNP	CHR	BP	A1	A0	Parents' attained age	Parents' age at death	Both parents top 10%	Mothers' attained age	Fathers' attained age	A1F	Beta	SE	Implicated gene(s)
***Combined parents' age results***															
	**Common & in GWAS catalogue of findings**														
		rs602633	1	109821511	T	G	**2.7E-08**	2.9E-03	2.4E-03	2.1E-03	3.6E-09	0.217	−0.0150	0.0027	*CLESR2 … PSRC1*
		rs28383322	6	32592796	C	T	**5.3E-11**	9.8E-03	2.0E-04	3.5E-09	2.0E-04	0.783	0.0182	0.0028	*HLA-DRB1… HLA-DQA1*
		rs55730499	6	161005610	C	T	**1.7E-18**	5.4E-11	2.7E-08	5.5E-10	5.9E-17	0.920	−0.0361	0.0041	*LPA*
		rs1556516	9	22100176	G	C	**4.7E-16**	1.5E-10	2.5E-08	8.7E-05	1.3E-20	0.502	−0.0181	0.0022	*CDKN2B-AS1 (ANRIL)*
		rs7137828	12	111932800	C	T	**3.4E-14**	3.1E-08	3.2E-06	2.2E-07	2.7E-12	0.483	0.0170	0.0022	*SH2B3/ATXN2*
		rs1317286	15	78896129	A	G	**1.2E-26**	1.8E-11	4.0E-09	2.1E-07	1.5E-31	0.668	−0.0254	0.0024	*CHRNA3*
		rs17514846	15	91416550	C	A	**7.1E-10**	6.6E-07	2.0E-04	7.8E-04	1.2E-07	0.530	−0.0139	0.0023	*FURIN*
		rs429358	19	45411941	T	C	**1.4E-74**	5.0E-22	7.1E-46	1.3E-68	1.9E-28	0.845	−0.0566	0.0031	*APOE/APOC1*
	**Common**														
		rs1627804	6	107400428	C	A	1.1E-04	**4.0E-08**	4.5E-06	5.7E-03	2.4E-04	0.684	0.0197	0.0035	*BEND3*
		rs7844965	8	27442064	G	A	**7.7E-09**	1.5E-04	6.0E-04	4.9E-04	2.2E-07	0.768	0.0154	0.0027	*EPHX2*
		rs61978928	14	75321714	T	C	**2.0E-08**	7.7E-05	3.3E-07	1.0E-04	1.9E-07	0.688	0.0136	0.0024	*PROX2*
		rs28926173	18	13886719	G	A	6.4E-06	2.4E-05	**2.3E-08**	2.4E-04	2.5E-02	0.953	−0.0264	0.0047	*MC2R*
	**Rare (<2%)**														
		rs146254978	1	74867799	T	C	2.1E-02	**4.6E-08**	1.9E-03	4.3E-01	3.0E-04	0.980	−0.0692	0.0126	*FPGT*/*TNNI3K*
		rs139137459	11	119269958	G	A	5.3E-03	1.1E-01	**2.7E-08**	3.0E-02	3.1E-02	0.996	−0.0906	0.0162	*USP2-AS1*
***Loci only emerging in mothers' analysis***															
		rs3130507	6	31147476	G	A	3.5E-06	6.1E-02	8.9E-01	**2.1E-10**	2.4E-01	0.720	−0.0155	0.0024	*PSORS1C3 … [x]*
		13:31871514	13	31871514	T	G	5.6E-04	4.5E-01	7.0E-01	**4.7E-08**	3.4E-01	0.995	0.0979	0.0180	*B3GALTL*
		rs61949650	13	64836488	T	C	4.2E-05	1.5E-03	1.5E-03	**2.9E-08**	6.7E-01	0.938	0.0251	0.0045	*(intergenic)*
***Loci only emerging in fathers' analysis***															
		rs3131621	6	31425499	A	G	8.9E-08	1.9E-02	4.8E-01	3.5E-05	**3.6E-08**	0.629	−0.0124	0.0022	*MICA … MICB*
		rs13262617	8	59838133	A	G	9.6E-03	7.3E-03	1.3E-01	8.1E-01	**3.1E-08**	0.970	0.0360	0.0064	*TOX*
		rs61905747	11	113639842	A	C	3.6E-05	3.5E-02	7.0E-02	2.2E-01	**5.5E-09**	0.809	−0.0160	0.0028	*ZW10*
		rs74011415	15	47660194	G	A	7.0E-06	3.9E-02	2.6E-02	5.0E-02	**1.4E-08**	0.881	−0.0192	0.0034	*SEMA6D*
		rs12461964	19	41341229	A	G	1.8E-04	1.5E-01	7.8E-01	4.8E-01	**8.2E-09**	0.504	−0.0126	0.0022	*EGLN2 … CYP2A6*
		rs74444983	19	45745607	T	C	4.1E-05	9.1E-03	1.1E-01	6.9E-02	**9.1E-09**	0.741	−0.0143	0.0025	*EXOC3L2 … MARK4*
		rs6108784	20	10964366	T	C	7.7E-04	4.3E-01	5.7E-02	7.8E-01	**1.2E-10**	0.586	−0.0143	0.0022	*C20orf187*
		rs2273500	20	61986949	T	C	4.4E-07	8.9E-02	1.8E-01	3.8E-03	**1.5E-08**	0.856	−0.0176	0.0031	*CHRNA4*

Lead SNP = SNP with lowest p-value in 1Mb region

BP = Genomic position, build 37 (hg19), A1F = frequency of A1, the effect allele, P = BOLT LMM p-value (A1F, BETA, and SE are for p-value in bold), SE = Standard Error

Beta = A1 effect on outcome. For attained age (Martingale residuals) a negative BETA = reduced hazard, i.e. increased attained age

Implicated gene(s) = Variants in locus usually intronic, or exonic (indicated by underlining). If two genes separated by dots, the SNP is intergenic

Associations with *APOE* related rs429358 (p=5×10^−22^) variant were confirmed, as were associations with the *CHRNA3/5* (nicotinic acetylcholine receptor) locus (rs1317286, 33.2% minor allele frequency, p=1.2×10^−26^). Associations were also found for six other loci already implicated in disease specific GWAS; the strongest association was with rs55730499 (8% frequency, p=1.7×10^−18^) in an intron of *LPA* gene, associated with lipoprotein A and LDL cholesterol levels, plus CAD [14]. Variants in the 9p21 region, which includes *CDKN2B-AS1* (*ANRIL*) and *CDKN2A/B*, were also associated (lead SNP rs1556516, 49.8% frequency, p=4.7×10^−16^); this locus is involved in cellular senescence and associated with myocardial infarction and peripheral vascular disease [14]. Genetic variants across the 9p21.3 locus have previously also been associated with type-2 diabetes and cancers [14] and in longevity/survival analyses (see below), but only the CAD-associated loci were associated (p<5×10^−8^) with parent's lifespan (Figure 2; Supplementary Table 2).

Associations with parents' attained age were also present for rs7137828 (48.3% frequency, p=3.4×10^−14^) near *ATXN2*, coding for Ataxin-2 which is involved in endocytosis and is associated with blood traits and autoimmune disease [14]. SNPs in LD (R^2^=0.91) with rs7137828 include rs3184504 (48% frequency, p=4×10^−8^), a missense variant in the *SH2B3* gene, associated with blood pressure, maternal birth weight, TNF-α levels, colorectal cancer, Inflammatory Bowel Disease, LDL cholesterol levels and stroke [14]. Other known loci included rs28383322 (located between *HLA-DRB1* and *HLA-DQA1*, p=5.3×10^−11^), previously associated with atopic dermatitis; rs17514846 (located in an intron of *FURIN*, p=7.1×10^−10^), previously associated with blood pressure; and rs602633 (located between *CLESR2* and *PSRC1*, p=2.7×10^−8^), previously associated with CAD and LDL [14].

Two associations were discovered for novel variants not previously linked in GWAS to other traits or diseases; rs7844965, located in an intron of *EPHX2* (23.2% frequency, p=7.7×10^−9^), and rs61978928, located in the 3′ UTR of gene *PROX2* (31.2% frequency, p=2×10^−8^).

### A parents' attained age genetic risk score in independent cohorts

We calculated a genetic risk score (GRS) combining the 10 attained parental age associated variants, weighted by the estimated effect sizes. Genotyped samples of European descent from the Health and Retirement Study (HRS, *n*=12,940 in genetic analyses) and the Wisconsin Longitudinal Study (WLS, *n*=8,952 from 6,683 families) were too small to test individual variant associations: the sample size needed for 80% power to detect an association between the *APOE* variant and parents' lifespan at p<0.05 (R^2^=0.0005 in UK Biobank) is 15,598, but much larger samples were required for the smaller-effect variants discovered in UK Biobank. We therefore tested the parents' attained age GRS; the sample size needed for an 80% chance of detecting an association of this magnitude (R^2^=0.002 in UKB) with p<0.05 was 3,924. In Cox's proportional hazards survival models against both dead and alive parents attained ages in HRS (*n*=12,345, number of events=9,809), increasing GRS was associated with longer survival (mortality Hazard Ratio=0.961, 95% CIs=0.942 to 0.980, p=6×10^−5^, adjusted for offspring age, sex, and genetic principal components 1-5). In the WLS (*n*=7,437, number of events=6,683, errors clustered by family), increasing GRS was associated with longer survival (mortality Hazard Ratio=0.963, 95% CIs=0.938 to 0.988, p=4×10^−3^, adjusted for age at saliva collection, sex, and genetic principal components 1-5).

### Functional implications and pathways analysis

We investigated effects of the parents' attained age SNPs on expression of genes in multiple tissues, such as whole blood [15], using the FUMA tools [16]. Expression of 53 genes were associated, including 8 of the 10 nearest genes to the variants identified above (Supplementary Table 5 & 6).

In MAGENTA gene-set analysis, only two pathways were significantly enriched after correction for multiple testing, both related to nicotinic acetylcholine receptor binding and downstream events (Supplementary Table 4).

### Sensitivity analyses of combined parents' attained age analyses

We generated QQ plots for all GWAS (Supplementary Figure 2) and observed some genomic inflation (Lambda GC for parents' attained age = 1.199), consistent with human longevity being highly polygenic, but perhaps also representing some effect of relatedness in inflating results. The BOLT-LMM software used for analysis accounts for relatedness in the methods, but we investigated further; the 10 variants associated with attained parental age were tested in Cox proportional hazards (CoxPH) models against parents' attained age, to assess whether the associations observed with rank-normalized Martingale residuals were robust. These models also excluded related (≥3^rd^-degree) participants, leaving a reduced sample size of n=326,864, including n=153,101 with censored data due to at least one parent still being alive. Five of the 10 loci remained genome wide significant (p<5×10^−8^) in these models; *APOE*, *CHRNA3*, *LPA*, *ANRIL*, and *ATAXN2/SH2B3* (Supplementary Table 3) with the remaining five directionally consistent and associated at p≤3.1*10^−6^.

**Table 3:** Variants implicated in disease and trait GWAS that were associated with parents' attained age at near genome wide significance (p>1×10^−8^ but p<1*10^−5^)

SNP	CHR	BP	A1	A0	A1FREQ	BETA	SE	P	Gene	Previous associations with locus
rs11552449	1	114448389	C	T	0.826	−0.0134	0.0030	6.3E-06	*DCLRE1B*	Breast Cancer [18]
rs6689306	1	154395946	A	G	0.419	0.0111	0.0023	1.5E-06	*IL6R*	CAD [40]
rs11126666	2	26928811	G	A	0.745	−0.0117	0.0026	4.5E-06	*KCNK3*	BMI [17]
rs7593947	2	60704933	A	T	0.526	−0.0110	0.0023	1.2E-06	*BCL11A*	Educational attainment [19]
4:77410318	4	77410318	C	A	0.544	0.0100	0.0022	9.4E-06	*SHROOM3*	Red blood cell count [41]
rs1986734	4	77420784	C	T	0.476	0.0101	0.0022	6.9E-06	*SHROOM3*	Creatinine [42]
rs806794	6	26200677	A	G	0.728	0.0115	0.0025	5.0E-06	*HIST1H2BF … HIST1H4E*	Height [43]
rs4014195	11	65506822	C	G	0.656	−0.0108	0.0024	3.7E-06	*RNASEH2C … AP5B1*	eGFR [20]
rs633185	11	100593538	G	C	0.285	−0.0112	0.0025	8.9E-06	*ARHGAP42*	DBP, SBP [44]
rs56289821	19	11188247	G	A	0.882	0.0155	0.0035	7.3E-06	*SMARCA4 … LDLR*	CAD [40]
rs6511720	19	11202306	G	T	0.881	0.0165	0.0035	1.8E-06	*LDLR*	LDL [45]
rs17724992	19	18454825	A	G	0.733	0.0124	0.0025	8.6E-07	*PGPEP1*	BMI [17]

BP = Genomic position, build 37 (hg19), BETA = Effect on outcome with respect to A1 (the effect allele), P = BOLT LMM p-value

**Figure 1:**
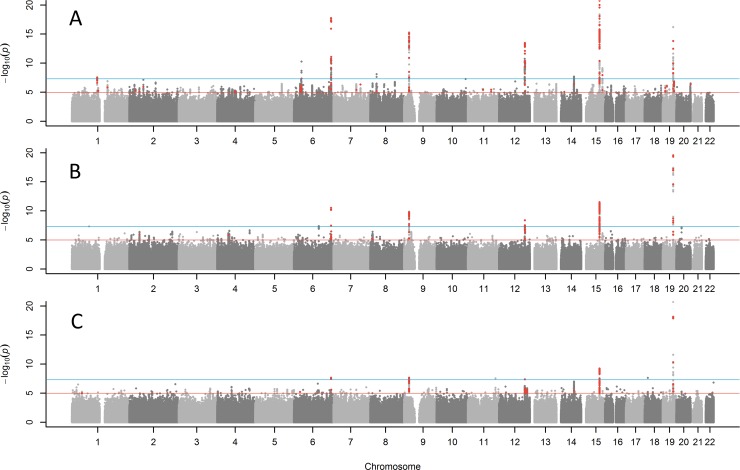
Manhattan plots for GWAS of parent's lifespan **(A)** combined parents' attained age GWAS including living parents, (**B**) combined age at death of mother and father, (**C**) both parents reached top 10% of survival (mothers reached ≥90 years, father reached ≥87 years). Red points are variants (p<1×10^−5^) previously associated with other traits. The blue line indicates p=5×10^−8^. The Y-axis has been capped at 20 (p=1×10^−20^) to aid in visualizing the results.

We performed sensitivity analyses only including parents who had died (Figure 1B; Table 2), as the ages of living parents at the UKB study baseline are clearly an incomplete measure of their longevity. This analysis included a smaller sample size of n=208,118 UKB participants with both parental ages of death known. The same five of the ten loci reported above remained genome-wide significant (*APOE*, *CHRNA3*, *LPA*, *ANRIL*, and *SH2B3*) with the remaining loci remaining nominally significant. Two additional variants not identified in the previous analysis emerged as candidates for follow-up: rs1627804 (32% frequency, p=4×10^−8^), in an intron of *BEND3*, a transcriptional repressor which associates with the NoRC (nucleolar remodeling complex) complex and playing a key role in repressing ribosomal DNA transcription; and rs146254978 (2% frequency, p=4.6×10^−8^), located in an intron of both *FPGT* and *TNNI3K*.

### GWAS of both parents achieving longest 10% survival

We next tested binary associations with having both parents surviving to the longest 10% of lifespans, using sex-specific age-at-death cut-offs (father ≥87, mother ≥90, including living parents aged over these age cut-offs), to assess whether associations were attributable to longer lives rather than early deaths. This analysis confirmed associations (Figure 1C; Table 1) with variants in the *APOE*, *CHRNA3, LPA and CDKN2B-AS1*, with a suggestive association with *ATXN2* (p=3.4×10^−6^). Two further loci just reached significance in this analysis: rs28926173 (4.7% frequency, p=2.3×10^−8^) in an intron of gene *MC2R*, and rs139137459 (0.4% frequency, p=2.7×10^−8^) in an intron of *USP2-AS1* (near *USP2*). The *MC2R* variant was associated at p=6.4×10^−6^ with parents' attained age, but the USP2-AS1 variant had a low population frequency (0.4%) and was not significant in the continuous trait analyses.

### Genetic risk score associations with centenarian status and offspring health status in UKB

There were 691 UKB participants with a centenarian mother (the longest lived 0.1% of maternal deaths in the sample), and 747 with a father aged ≥98 years (the corresponding age cut-off of longest lived 0.1%); only five participants had both parents in the longest lived 0.1%. Although this relatively small group (0.3% of the full attained age sample) were included in the discovery analysis, we tested the GRS for association with having a centenarian parent (in the un-related UK Biobank participants, *n*=1,181), against controls whose (both) parents died before age 80 (*n*=66,280). Increasing attained age GRS was associated with increased odds of having a centenarian parent (OR per standard deviation of GRS=1.193, 95% CIs=1.130 to 1.260, p=3.0×10^−10^).

### GWAS of mothers' and fathers' age at death separately

We tested for parental gender differences by conducting separate GWAS for mothers' and fathers' attained ages (including living parents). Eleven additional genetic variants were identified, three only associated with mother's age at death, eight associated with father's age at death (Table 2). Results included variants previously associated with the autoimmune diseases psoriasis and idiopathic membranous nephropathy (for mothers), and an independent nicotine-dependence locus on chromosome 20 for fathers. Novel gene-longevity associations included genes *MICA/B* (a locus previously linked to autoimmune conditions rheumatoid arthritis and multiple sclerosis), *TOX*, *ZW10*, *SEMA6D*, *EGLN2/CYP2A6*, *EXOC3L2/MARK4* and *C20orf187*.

### Comparison with recently reported GWAS analyses of longevity

A recent large analysis by Joshi et al. [2], with up to 294,998 participants in their analysis of European parents' attained age, included the first third of UK Biobank genotyping data plus 26 other cohorts. For survival past age 40 years, associations were confirmed for *APOE* and *CHRNA3/5*, plus novel associations with overall longevity (deaths or survival to age 40 years and over) with *HLA-DQA1/DRB1* and *LPA*, and with replication of *CDKN2A/B* and *SH2B3.* While these results overlap with our attained age analysis, they also reported an association with *FOXO3A* variants, but we found no significant associations for this locus (p>0.01) (see Supplementary Table 2 for details). No analyses of longer or extreme survival were reported.

McDaid et al. [6] undertook a Bayesian association scan of general lifespan (in the first third of UK Biobank), weighted for 58 disease related GWAS studies, and identified 16 individual variants at genome wide significance. Of these, four (*LPA, CDKN2BAS, CHRNA5* and *APOC1*) reached conventional GWAS significance in our analyses, and *CELSR2* only reached significance for mothers' age at death.

While the above studies focused mainly on overall lifespan, for aging research survival to extreme ages is of special interest. In a powerful study of extreme longevity, Fortney et al. used samples including 801 centenarians and 5406 subjects aged 90 plus. Disease GWAS results were used to weight genetic variants, using a false discovery rate of 10% [5], at which associations with *APOE*, *SH2B3/ATXN2*, *ANRIL*, *LPA*, and *HLA* were reported (although only *APOE* reached conventional genome wide significance). Fortney et al. also identified variants at *KCNT2* and *FADS1, which* were not associated with parents' attained age in our analysis, with the *ABO* SNP (rs514659) only just reaching nominal significance (p=0.015).

A previous meta-analysis comparing older individuals (age 80 or 90+) to younger controls reported a genome wide association at the 5q33.3 locus, but no significant associations were found for the other two loci (p>0.01). Various other variants, including in mitochondrial DNA, have been suggested as associated with longevity. In our GWAS we tested associations with mitochondrial variants, plus variants on the X and Y chromosomes, using the directly genotyped microarray data: none were significantly associated (p>1×10^−5^) with the studied phenotypes.

### Disease-associated genetic variants; candidates for future work

We investigated genetic variants associated with parents' attained age (p<1×10^−5^) that have previously been associated with other traits for promising candidates for future work and identified 12 independent signals (Table 3). These included SNPs associated with BMI (rs11126666, [17]), breast cancer (rs11552449, [18]), educational attainment (rs7593947, [19]), and kidney function (estimated Glomerular Filtration Rate, rs4014195, [20]), in addition to a variant in *IL6R* (rs6689306) associated with CAD.

## DISCUSSION

In this analysis we aimed to extend our previously published GWAS study of parental longevity to include the newly available full UKB data, achieving an unprecedented sample size for longevity studies. We have confirmed associations with *APOE* and *CHRNA3* variants, and additionally identified 8 novel genome wide significant variants for our principal phenotype of combined parents' attained age, plus several additional variants for our related longevity phenotypes. Overall, the findings suggest that human longevity is a polygenic trait, influenced by large numbers of modest effect variants. Many of the variants identified have been previously implicated in age-related diseases or traits, notably with coronary artery disease and lipid levels. Several loci are near genes involved in inflammation, an important pathway in the development of biological aging [21]. In additional analyses of mothers' and fathers' attained ages separately we identified 11 additional genetic variants, three only associated with mother's age at death and eight associated only with father's age at death.

**Figure 2:**
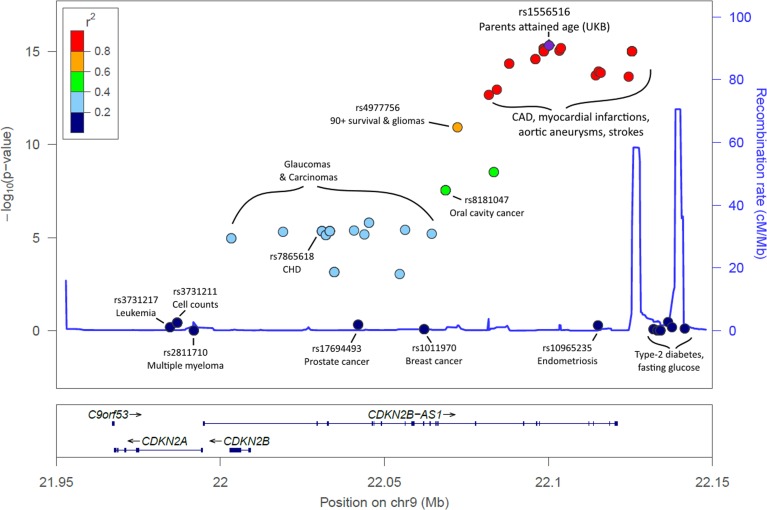
LocusZoom plot of the *CDKN2A/B/ANRIL* gene cluster and known disease variants Known diseaseassociated genetic variants have been highlighted in the 9p21.3 region, in addition to the lead SNP identified in our analysis. Association p-values indicate the strength of association with parents' attained age. The variants known to influence coronary artery disease (CAD) and related-traits are those most-associated with parents' attained age. Variants previously associated with cancer are markedly less associated with parents' attained age, with type-2 diabetes variants not associated (p>0.05). Details, including references, are included in Supplementary Table 2.

Parental lifespan-associated variants included rs1556516, located in an intron of *CDKN2B-AS1* (*ANRIL*), a long non-coding RNA; variants in this region, 9p21.3, have previously been associated with CAD, type-2 diabetes, and cancer [14]. *ANRIL* is known to regulate neighbor tumour suppressors *CDKN2A* (encoding proteins p14arf and p16) and *CDKN2B* (encoding protein p15) epigenetically through directing polycomb gene regulators to modify the chromatin state [22]. *ANRIL* therefore plays a key role in regulation of cell proliferation and senescence. It is interesting to note that removal of CDKN2a expressing senescent cells achieved significant rescue of aging phenotypes in mice [23]. Intriguingly, the variants in 9p21 associated with lifespan are predominantly related to arterial disease (coronary and peripheral arterial disease), rather than those influencing cancer or type II diabetes risk (Figure 2). Decreased expression of specific *ANRIL* isoforms is reported to affect expression of *ADIPOR1* (adiponectin receptor with a role in fatty acid catabolism and inflammation), *VAMP3* (membrane protein with a role in phagocytosis) and *C11orf10* (in proximity to genes associated with metabolic syndrome) [24]. The lifespan-increasing allele for the lead SNP (rs1556516-G) is known to increase expression of *ANRIL* [25]; atherosclerosis-related phenotypes are associated with lower expression of *ANRIL*, suggesting a causal mechanism of action for this SNP on lifespan.

rs11065979 (chromosome 11) is located near the *ATXN2* gene, which is known to be associated with autoimmune disease and blood traits [14]. The variant is also highly correlated (R^2^=0.91) with an exonic variant in *SH2B3*, previously linked to CAD, blood pressure, TNF-α, cell counts and colorectal cancer [14]. *SH2B3* encodes the lymphocyte adapter protein (LNK), which plays a key role linking inflammation and hypertension, particularly in the kidney and blood vessels [26].

*EPHX2* is a hydrolase involved in the degradation of vasoactive and anti-inflammatory acids, with possible function in LDL-cholesterol metabolism [27]. Exonic polymorphisms in *EPHX2* are associated with coronary artery calcification [28]. *PROX2* is a transcription factor of unknown function implicated in cardiac muscle and neuron differentiation by gene ontology [29]. Genetic variants in *FURIN*, a gene located in chromosome 15, were previously associated with blood pressure and are here associated with parents' attained age; the protein is a widely-expressed membrane-bound protease, and is essential for regulatory and effector T-cell function [30].

Other novel genes implicated include *MC2R*, where the rare allele is associated with 15.7% increased likelihood of both parents reaching the top 10% of survival. This gene encodes the adrenocorticotropic hormone receptor, involved in the secretion of cortisol. This pathway is key to the stress response, which is regarded as a pillar of the aging process [31]. Lack of this functioning receptor can result in chronic hormone-imbalance and various conditions, including subclinical Cushing syndrome, plus impaired blood sugar regulation and immune system function [32].

For four of the novel parents' attained age-associated variants it is the rare allele that is associated with increased lifespan; rs602633 near *CLESR2* and *PSRC1*; rs28383322 near *HLA-DRB1* and *HRL-DQA1*; rs7844965 in *EPHX2*; and rs61978928 in *PROX2*. This is unusual, as for the other genetic variants it is the common allele that confers the advantage (the rare variant tends to be associated with increased disease-risk, such as CAD).

We have shown that a genetic risk score based on the 10 variants associated with combined parents' age of death is associated with parental survival in two independent US aging cohorts, suggesting that our overall findings are robust. In addition to the more common variants identified (i.e. minor allele frequency >1%) there were several rarer variants, such as rs139137459 in *USP2-AS1* (0.4% frequency): these associations are less certain, as the GWAS significance level of p<5*10^−8^ may not be sufficiently rigorous for testing low frequency variants. The identified SNPs previously linked to GWAS traits (such as a variant in *IL6R*) have a high prior probability and are especially likely to be robust.

This study is limited to European ancestry (white British) UK Biobank participants, and results may not be applicable to other populations. UK Biobank is a healthier volunteer study with a sample that was less likely to be obese, to smoke or to drink regularly, compared to the general population [33], perhaps reducing our power to detect variants associated with health behaviors, although the sample still contains many participants who were exposed to these factors. We were not able to verify reported parental ages or ages of death, perhaps introducing misclassification. Also, ages of birth of deceased parents were not recorded, so it is not possible to compare study and national cohort survival.

We are limited by the coverage of the available genotyping microarray, and have used data on imputed SNPs from the Haplotype Reference Consortium panel, with imputation using the UK10K reference panel to follow. As with all GWAS discovered loci, the identification of affected genes is not straightforward, but we have followed the convention of highlighting the nearest genes, which for 8 of the 10 attained age variants was confirmed by the eQTL data.

We studied the normal range of parental ages at death, as well as the top end of longevity (top 10% of survival), to establish whether associations were driven by early deaths. Our longest lived 10% analysis is similar in age (87+ years for fathers, 90+ for mothers) to those studied in many of the previous GWAS of longevity [3,4]. Analyses with bigger samples of those attaining extreme ages are needed, but unfortunately our sample with extreme surviving parents (e.g. centenarians) is too small for GWAS analyses.

In addition to these limitations, our analysis has several strengths. Our sample size yielded unprecedented power to detect variant associations: indeed the power (>99% to detect an allele of 1% minor allele frequency accounting for 0.1% of phenotype variance) is sufficient to suggest that we have identified all moderate to larger effect common genotyped or imputed variants in our studied population. Our analyses were undertaken in a single cohort: previous longevity GWAS combined data from diverse cohorts, which has been suggested as explaining difficulties in detecting and replicating longevity variants [34].

### Future work

Replication and extension of the novel associations in other large cohorts and in different ancestries is needed, especially for variants narrowly achieving genome wide significance. The associations identified should be examined in future studies of age-related traits and disease. More work is needed to characterize the variants identified for biological functions; this could include epigenetic and gene expression studies, such as analysis of allele-specific expression imbalance in heterozygote individuals to determine the gene of action for genetic variants [35]. The variants identified in our analysis may provide useful information for designing targeted prevention or precision treatments to promote human longevity, perhaps particularly related to targets in inflammatory pathways and cardiovascular disease.

## CONCLUSIONS

Our analysis of parental longevity involved the largest sample size thus far achieved, with ample power (>99%) to detect even modest associations. The results indicate that human longevity is a polygenetic trait, including likely prominent effects involving cell senescence, inflammation, autoimmunity and the stress response, plus lipid levels and vascular disease. The results suggest that the identified variants are involved in achieving long lifespans, and are not limited to causing early deaths. The results also suggest that there may be gender differences in routes to longevity, with autoimmune variants being particularly important in women. Overall these results are consistent with some previous analyses in humans and with certain key hallmarks or pillars of the biology of aging. The variants identified may be useful for identifying targeted prevention and precision treatments for extending human lifespans.

## METHODS

### Parental age at death and longevity phenotypes

Participants were asked the age at which their parents had died (or their current age if still alive). Analyses were performed separately on mother's age at death and father's age at death, and also on a combined phenotype. To reduce the effect of higher ages at death of mothers (compared to the fathers) we first z-transformed the mothers and fathers age at deaths before combining the z-scores into a single summed phenotype. Offspring of parents who died prematurely were excluded because the cause of death of the participant's parents was not asked, so we could not exclude accidental deaths explicitly. To determine the age at death considered to be too young to be part of the normal distribution of age-related mortality, we used previously described methods to define a normal distribution of ages at death for mothers and fathers separately, as described previously [9]). In brief, non-linear least square regression models were used to fit a normal curve on age at death data from the modal (M) age at death to the oldest ages; the younger (left) half of the curve is then imposed, to avoid the severe skewing of the distribution caused by the excess of deaths below the modal age. The analysis identified the following early death cut-points (based on modal age at death minus 1 Standard Deviation): mothers 57 years, fathers 46 years.

In addition, combined parents' age (including alive parents) were derived from Cox's proportional hazards regression models using Martingale residuals, as described by Joshi et al. [13]. Events were defined as the both parents having died. We also defined a binary phenotype by determining the top 10% of age at death for mothers (≥90 years) and fathers (≥87 years) separately. Controls were participants where both parents died before the age of 80. We were able to increase the number of “long lived” parents by including alive participants beyond the cut-off.

### UK Biobank

Between 2006 and 2010, 503,325 volunteers (aged 45 to 69 years old) were recruited from across the United Kingdom to the UK Biobank study [36]. Of these, 389,166 participants met the inclusion criteria for at least one analysis: participants with complete genetics data and either date of death of their parents or their current age if alive. Participants were excluded if they reported themselves as adopted, or whose parents died more than 1 standard deviation below the modal age of death (mothers <57 years, fathers <46 years). Several longevity phenotypes are defined below based on the age at death of the participant's parents.

Genetic data was available on 488,377 UK Biobank participants after genotype calling and quality control performed centrally by the UK Biobank team [37]. We selected 451,447 participants identified as ‘white European’ through self-report and verified through principal components analysis based on genotypes. Briefly, principal components were generated in the 1000 Genomes Cohort using high-confidence SNPs to obtain their individual loadings. These loadings were then used to project all of the UK Biobank samples into the same principal component space and individuals were then clustered using principal components 1 to 4. Related individuals were identified through kinship analysis, although these participants were included in genome-wide analysis using BOLT-LMM (see below), those related to the third-degree or closer were excluded in sensitivity analyses.

Imputation of 39,235,157 genetic variants from the Haplotype Reference Consortium panel was performed using IMPUTE4 centrally by the UK Biobank team [37]. After filtering for variants with MAF ≥0.1%, missingness <1.5%, imputation quality >0.1 and with Hardy-Weinberg equilibrium (HWE) P>1×10^−6^ within the white British participants 11,516,125 imputed autosomal variants were eligible for the analyses.

We also utilized data directly from the microarrays for variants on the X (n=19,381) and Y (n=284) chromosomes, and on the mitochondrial genome (n=135), which were unavailable in the imputed dataset.

### Health and Retirement Study (HRS)

In HRS v2 data, phase 1-3 samples were combined (n=15,708) and SNPs were kept if no discordant genotype call among duplicate samples (2,315,518 SNPs). Related or duplicated participants and geno-typing controls were removed, as well as those with a missing call rate equal to or greater than 2%. After the sample quality control, 15,454 samples were kept and 84% of them (n=12,940) were of European descent.

2,075,208 out of 2,315,518 SNPs passed the composite filters by the University of Washington (Hardy-Weinberg equilibrium p-value < 1e-4, missing call rate ≥ 2%, sex difference in allelic frequency ≥ 2% and sex difference in heterozygosity > 0.3) and had a minor allele frequency greater than zero. As recommended, SNPs with a minor allele frequency smaller than 2% further were removed. At the end, 1,437,608 SNPs were carried forward for SNP pruning. The purpose is to choose less correlated SNPs for principal components analysis.

The SNP pruning was performed following the procedure: a) consider a window of 50 SNPs, b) calculate LD between each pair of SNPs in the window, c) remove one of a pair of SNPs if the LD is greater than 0.5, d) shift the window 5 SNPs forward and repeat the procedure. 514,246 SNPs remained after the SNP pruning and were used to run principal components analysis. As a result, the top 10 principal components were used to adjust for ancestry in a regression model. Both SNP pruning and principal components analyses were performed in PLINK1.9

The samples in the genetic data were mapped to the phenotype data of HRS to retrieve parental longevity phenotypes. We used the phenotype data from the most recent wave or visit of HRS where parental data was missing if the parent died before the last visit. To recover the data, we sequentially looked back at the last visit until the parental data was available. Father's and mother's data therefore could be from different visits and the participant's age varied with phenotypes. Mothers dying before 61 years and fathers dying before 46 years were excluded from the analysis, based on the same analysis of distribution of ages of death applied in UK Biobank. Parent's age was set to missing if he or she was still alive and younger than the child's age +10.

The genetic risk score analysis was conducted in a Cox regression model (against combined parents' attained age, alive or dead: derived by summing the z-transformed mother's and father's attained ages) with adjustment for age and sex of the participant and the top 10 principal components.

### Wisconsin Longitudinal Study (WLS)

In the WLS saliva samples were collected by mail in 2006-7 and during in-person interviews in 2011 for those who did not mail back samples. Genotyping on the Illumina HumanOmniExpress array was completed at Johns Hopkins' Center for Inherited Disease Research (CIDR) and data cleaning and imputation was performed in collaboration with the Genetic Analysis Center at the University of Washington. After quality control, there are a total of 9,012 respondents and 688,596 SNPs available for analysis. The sample has 4,601 singletons and 2,263 families with more than one member genotyped. Only 66 respondents are not of European descent.

Imputation of 33,549,349 genetic variants from the 1000 Genomes Project reference panel (Phase 3) was performed using IMPUTE2, with 686,143 study SNPs serving as the imputation basis. Of the 10 variants used to construct the GRS, two were directly genotyped and eight were imputed.

Parent's age was constructed by subtracting the year of birth from the year of death. When the latter was not available, the year of the most recent interview was used instead and the parent was classified as not dead. Mothers dying before 56 years and fathers dying before 46 years were excluded from the analysis, based on the same modelling of distributions of ages of death applied in UK Biobank. Parent's age was also set to missing if the disagreement between sibling reports exceeded 10 years. The analysis phenotype was the standardized sum of both parents' ages and was set to missing if either element was not available.

The genetic risk score analysis was conducted in a Cox regression with censored data, adjusting for age at saliva collection, sex of the participant and the top 5 principal components. Standard errors were clustered at the family level. Limiting the sample to the primary WLS respondents had a negligible effect on the results.

### Power Calculations

R package ‘pwr’ and function ‘pwr.f2.test’ was utilized for power calculations of regression analysis. The numerator degrees of freedom was set to 1, significance level to 0.05, and power to 0.8.

### Genome-Wide Association Study

We used BOLT-LMM v2.2 to model the associations between imputed variants (dosages) and each phenotype [38], which uses a linear mixed effects model approach. We looked at the results for variants with imputation quality >0.1, HWE p-values >1×10^−6^ and minor allele frequencies >0.1% in the white/British subset used for all analyses. For variants on the X, Y and mitochondrial chromosomes only in the directly-genotyped data we used Plink (v1.9) [39] in linear (additive) or binary (fisher) models, as appropriate, adjusted for the same covariates as above including the first 5 principal components from FlashPCA.

### Pathways Analysis and Functional Annotation

We utilized FUMA (Functional Mapping and Annotation of Genome-Wide Association Studies) to investigate the genetic variants associated with parents' attained age [16]. In particular, FUMA performs gene-set analysis (using MAGMA) to identify pathways enriched amongst the significant genes (weighted by the SNP-associations in proximity to them). In addition, FUMA searched eQTL databases to identify SNPs that significant alter the expression of genes in various tissues.

## SUPPLEMENTARY MATERIAL FIGURES AND TABLES














