# Supplementary material for: Human longevity: 25 genetic loci associated in 389,166 UK biobank participants
Source: Aging (Albany NY). 2017 Dec 6;9(12):2504–20. doi: 10.18632/aging.101334 (PMC5764389; doi:10.18632/aging.101334)
Supplement: Supplementary file 1 [file aging-09-2504-s001.pdf]

SUPPLEMENTARY MATERIAL

Please browse the Full Text version to see Supplementary Tables related to this manuscript.

Supplementary Table 1. GWAS results – parents' age at death MARTINGALE. Significant SNPs (p<5x10-8) associated with parents' attained age.

Supplementary Table 2. Longevity candidate SNPs PDF. Extracted summary statistics for many candidate loci from previous publications related to lifespan.

Supplementary Table 3. CoxPH results of parents' attained age SNPs. Cox proportional hazards regression model results for the lead SNPs from the parents' attained age GWAS.

Supplementary Table 4. MAGENTA gene-set enrichment analysis. Results from the gene-set enrichment analysis from FUMA (MAGENTA methods).

Supplementary Table 5. FUMA eQTL analysis of parents' attained age-associated variants. SNPs associated with parents' attained age that are eQTLs - identified by FUMA methods.

Supplementary Table 6. eQTLs summarised. Summary of the eQTL association data from FUMA - expression of 53 unique genes affected by SNPs associated with parents' attained age.

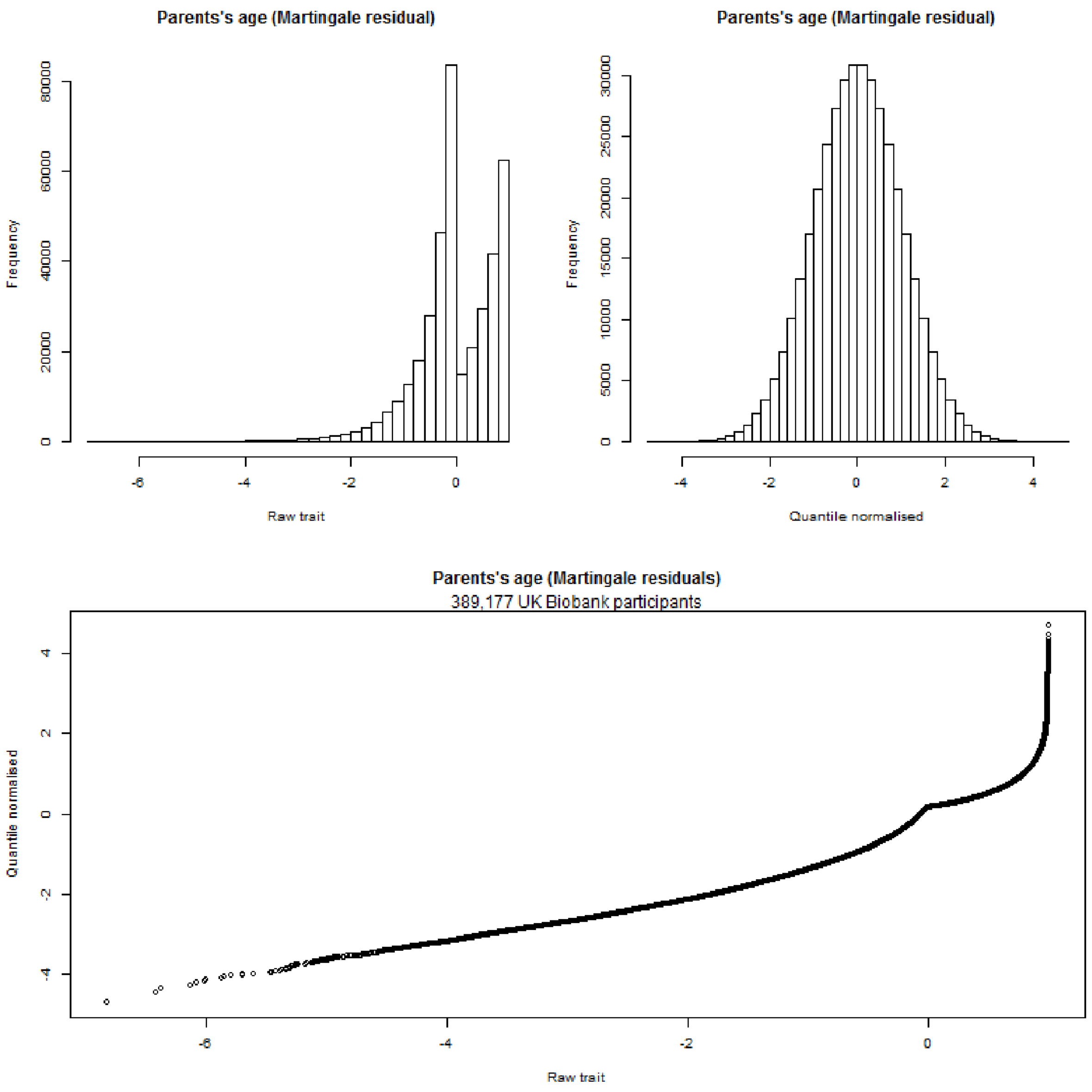

Supplementary Figure 1. Martingale residuals histograms. Histograms showing the raw and quantile normalised Martingale residuals.

1. Parents attained age (including both alive and dead) *n*=389,166
   Lambda GC = 1.199

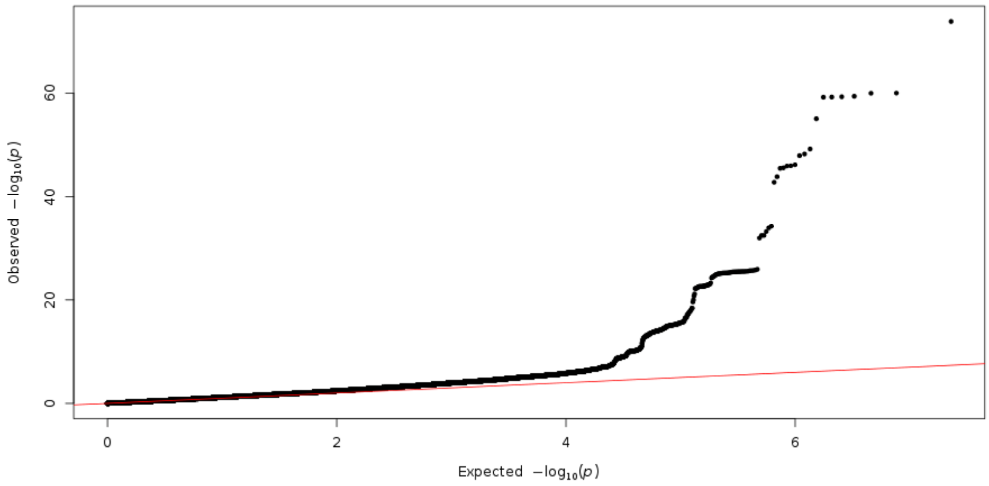

2. Parents age at death (only including parents who died) *n*=208,118
   Lambda GC = 1.147

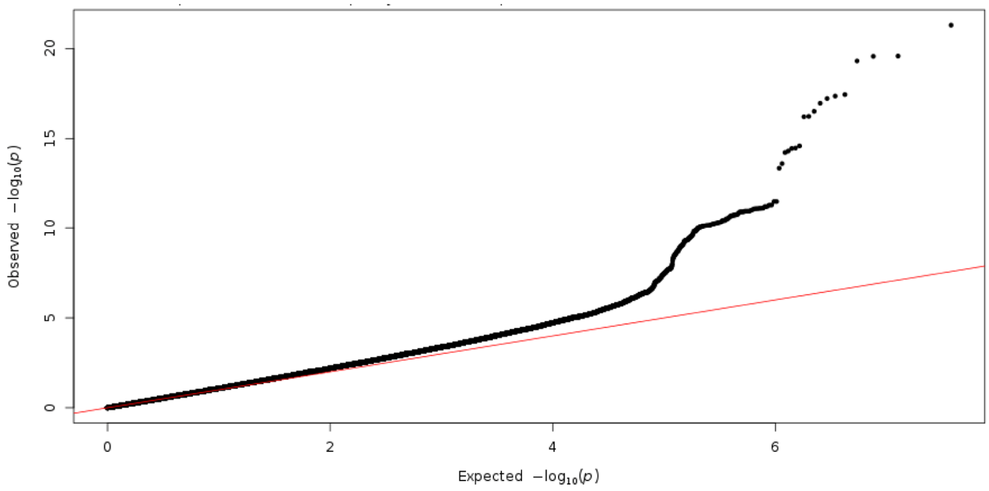

Supplementary Figure 2. GWAS QQ plots 1-2. QQ plots for each of the 5 GWAS performed.

3. Both parents reached top 10% of age-specific distributions (≥90 for mothers, ≥87 for fathers) *n*= 86,949

   Lambda GC = 1.097

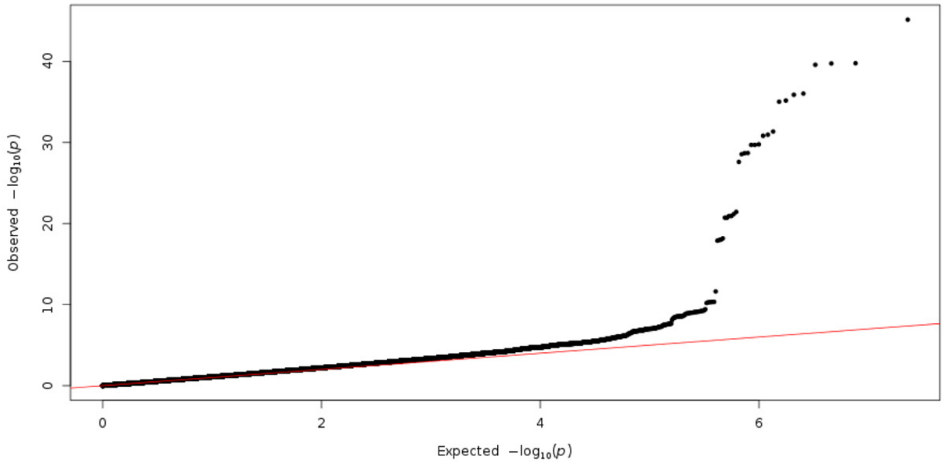

4. Mother's attained age (alive or dead) *n*=412,937

   Lambda GC = 1.147

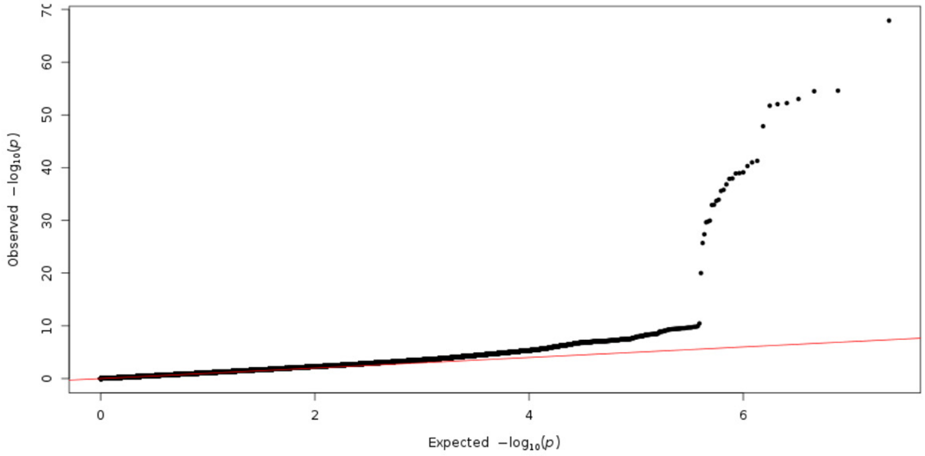

5. Father's attained age (alive or dead) *n*=415,311

   Lambda GC = 1.199

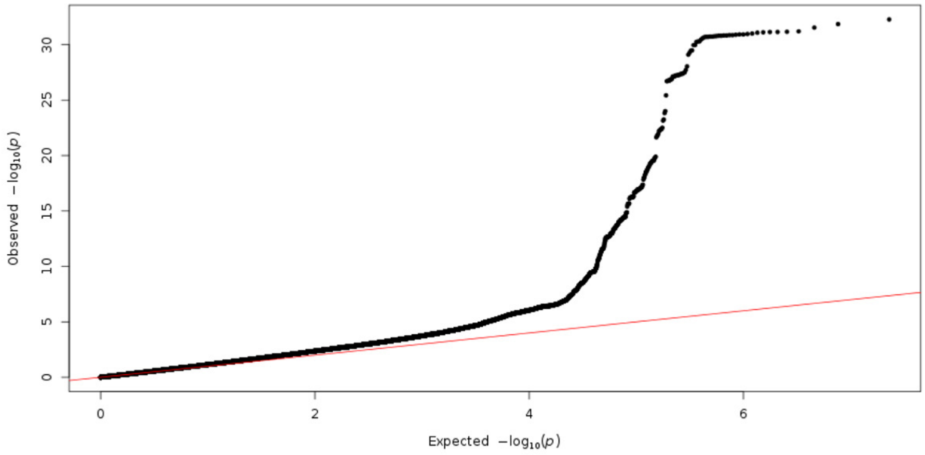

Supplementary Figure 2. GWAS QQ plots 3-5. QQ plots for each of the 5 GWAS performed.
